# P-2039. Prognostic factors of disease progression in patients with mild-to-moderate COVID-19 on early remdesivir treatment in Taiwan

**DOI:** 10.1093/ofid/ofae631.2195

**Published:** 2025-01-29

**Authors:** Yu-Chien Ho, Min-Chi Chang, Wen-Ying Lin, Chia-Ying Wu, Szu-Yu Liu, Chien Chuang, Chih-Han Juan, Chia-Jen Liu, Yi-Tsung Lin

**Affiliations:** Taipei Veterans General Hospital, Taipei, Taipei, Taiwan (Republic of China); Taipei Veterans General Hospital, Taipei, Taipei, Taiwan (Republic of China); Taipei Veterans General Hospital, Taipei, Taipei, Taiwan (Republic of China); Taipei Veterans General Hospital, Taipei, Taipei, Taiwan (Republic of China); Taipei Veterans General Hospital, Taipei, Taipei, Taiwan (Republic of China); Taipei Veterans General Hospital and National Yang Ming Chiao Tung University, Taipei, Taipei, Taiwan; Taipei Veterans General Hospital and National Yang Ming Chiao Tung University, Taipei, Taipei, Taiwan; Taipei Veterans General Hospital and National Yang Ming Chiao Tung University, Taipei, Taipei, Taiwan; Division of Infectious Diseases, Department of Medicine, Taipei Veterans General Hospital, Taipei, Taiwan, Taipei, Taipei, Taiwan (Republic of China)

## Abstract

**Background:**

An early 3-day course of remdesivir treatment was recommended for high risk patients with mild-to-moderate COVID-19. We investigated the prognostic factors of disease progression and mortality in patients with mild-to-moderate COVID-19 who were treated with a 3-day course of remdesivir in Taiwan.

**Figure:**
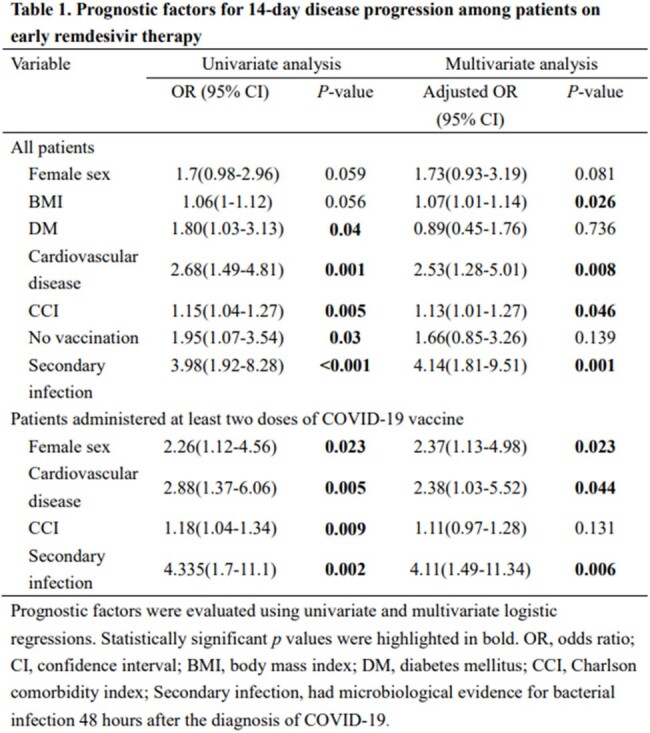

**Methods:**

Adult patients with mild-to-moderate COVID-19 treated with a 3-day course of remdesivir at Taipei Veterans General Hospital from April–July 2022 were identified. The main outcomes were 14-day disease progression (defined as increased oxygen requirement compared with the baseline condition or mortality) and 28-day mortality. Logistic regression was used to identify independent variables associated with poor outcomes.

**Figure:**
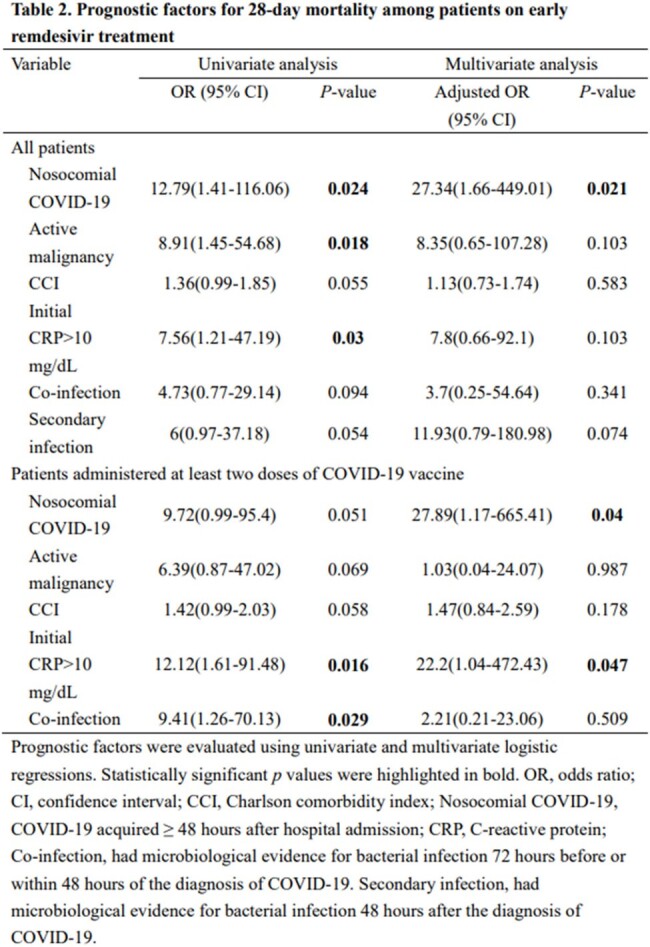

**Results:**

Among the 345 patients on early remdesivir treatment with a 3-day course, 62 patients (18%) had 14-day disease progression, and 5 patients (1.4%) died within 28 days. Eighty patients (23.2%) did not receive COVID-19 vaccine before the diagnosis of COVID-19. Body mass index, cardiovascular disease, Charlson Comorbidity Index, and secondary bacterial infection were independent factors associated with 14-day disease progression, and nosocomial COVID-19 was the only independent factor associated with 28-day mortality. In 233 patients (67.5%) administered at least two doses of COVID-19 vaccine, 38 patients (16.3%) had 14-day disease progression, and 4 patients (1.7%) died within 28 days. Female sex, cardiovascular disease, and secondary bacterial infection were independent factors for 14-day disease progression. Nosocomial COVID-19 and C-reactive protein levels >10 mg/dL were independent factors associated with 28-day mortality.

**Conclusion:**

Nosocomial COVID-19 and secondary bacterial infections predisposed these patients to poor outcomes, regardless of vaccination. Therefore, infection control measures are important in the fight against COVID-19.

**Disclosures:**

All Authors: No reported disclosures

